# A Colorimetric and Fluorescent Dual-Mode Sensor Based on a Smartphone-Assisted Laccase-like Nanoenzyme for the Detection of Tetracycline Antibiotics

**DOI:** 10.3390/nano15030162

**Published:** 2025-01-22

**Authors:** Hongyue Chen, Zining Wang, Qi Shi, Weiguo Shi, Yuguang Lv, Shuang Liu

**Affiliations:** 1 College of Pharmacy, Jiamusi University, Jiamusi 154007, China; 2 College of Basic Medicine, Jiamusi University, Jiamusi 154007, China

**Keywords:** copper nanoenzyme, laccase-like, fluorescent sensor, tetracycline antibiotics, multimodal detection

## Abstract

A copper-based nanoenzyme (Cu-BL) co-modified by L-L-lysine and 2-2-amino terephthalic acid has laccase-like activity and fluorescence characteristics. Based on this, a colorimetric and fluorescent dual-mode sensor was developed to visually and quantitatively detect tetracycline antibiotics (TCs), including tetracycline (TC), chlortetracycline (CTC), and oxytetracycline (OTC). In the colorimetric detection system, TCs can inhibit the generation of singlet oxygen (^1^O_2_) and weaken the ability of 2,4-dichlorophenol (2,4-DP) to be oxidized into pink-colored quinone substances. The linear ranges are 0.5–80 μM, 1–80 μM, and 0.25–80 μM, and the detection limits are 0.27 μM, 0.22 μM, and 0.26μM, respectively. In addition, due to the inner filter effect, tetracycline antibiotics can interact with Cu-BL, and with the increase in tetracycline antibiotic concentration, the fluorescence intensity will decrease. In addition, the smartphone sensing platform is combined with the colorimetric signal for the rapid and visual quantitative detection of tetracycline antibiotics. Generally speaking, the colorimetric/fluorescence dual-mode sensor demonstrates good stability, high specificity, and strong anti-interference capabilities, highlighting its practical application potential. This work is expected to offer novel insights for the development of multifunctional nanoenzymes and the integration of a multi-mode sensing platform.

## 1. Introduce

Antibiotic pollution is a global concern and significantly impacts human health, attracting a great deal of attention due to its potential health risks [1]. TCs, a class of broad-spectrum antibiotics including TC, CTC, OTC, etc., have been widely used in veterinary practice, animal husbandry, human treatment, and agricultural purposes for treating and controlling infectious bacterial diseases [2,3]. However, the overuse of TCs may lead to side effects, including allergic reactions, kidney failure, and the development of antibiotic resistance in humans [4,5]. In addition, the improper disposal of antibiotics can result in relatively high levels of antibiotics in the water environment [6]. Therefore, the accurate and specific detection of TCs will help eliminate their negative impacts.

Several quantitative analysis methods for TCs have been developed [7,8,9,10,11,12,13]. Although they have strong analytical parameters, they also have some obvious shortcomings. For example, the limitation of this very sensitive technology in practical use lies in the need for complex sample processing and skilled personnel, as well as their expensive equipment, expert operation, and complicated procedures. In addition, they are only in one detection mode and are easily affected by test conditions. In recent years, practical portable biosensors for detecting TCs have drawn significant attention. These biosensors are based on colorimetric or fluorescent detection with natural enzymes as the core components due to the high activity and selectivity of enzymes. However, natural enzymes are quite fragile and not applicable in complex environments. To address this limitation, nanoenzyme-based sensors have emerged. Thanks to their advantages like good stability, high activity, easy preparation, convenient operation, and low cost [14,15,16], they are widely used in the detection of drug molecules [17], heavy metal ions [18], and tumor markers [19], as well as in cancer treatment [20] and anti-bacteria [21] applications. Inspired by the structure of laccase, most studies use copper as the core element of the active substance and combine it with appropriate ligands to catalyze substrates by simulating the multi-copper coordination environment [22,23,24,25]. For example, Wang et al. [26], based on the peroxide activity of Fe_3_O_4_@Cu nanoparticles and “sandwich” oligonucleotide hybridization technology, developed a new type of tetracycline colorimetric sensor with high sensitivity and successfully applied it to the high-sensitivity detection of TC in milk samples. Wu et al. [27] used copper chloride dihydrate as the copper source, sodium borohydride as a reducing agent, and β-cyclodextrin as a protective agent to synthesize copper nanoenzymes (CuNZs) with good laccase activity by a grinding method. A rapid and simple OTC colorimetric determination method was established by using the laccase-like activity of CuNZs.

Colorimetric measurements usually monitor the change in absorbance, which is caused by the change in the concentration of the analyte at a specific wavelength. They have attracted attention due to their simplicity, low cost, and visualization of the processes involved. Similarly, fluorescence sensing techniques rely on spectrum fluorescence changes brought on by the contact of the probe and the detector. These techniques are quick, easy, sensitive, and reasonably priced. A dual-mode strategy of colorimetry and fluorescence is created by integrating two optical components into the sensor [28]. By establishing a cross-reference mechanism between colorimetric and fluorescence detection modes, this method effectively minimizes environmental interference and inherent errors [29,30]. Meanwhile, the deep integration with smart devices such as smartphones will make the detection process more portable and intelligent, providing more possibilities for immediate detection and on-site analysis [31], thus making greater contributions to safeguarding human health and promoting social development.

**Scheme 1:**
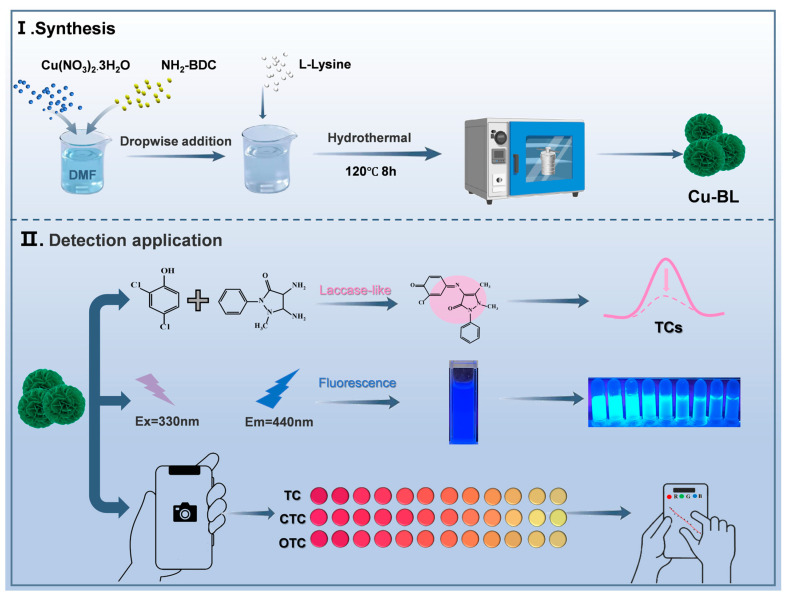
The synthesis of the laccase mimic Cu-BL and the principal diagram of the multimodal sensor used for the detection of TCs.

In this work, a Cu-BL-based dual-mode sensor for colorimetry and fluorescence (Scheme 1) was constructed for the detection of TCs. An L-lysine modified Cu-NH_2_-BDC material with laccase-like activity and capable of emitting blue fluorescence at 440 nm was prepared through a one-step solvothermal method. In the colorimetric detection system, due to its laccase-like catalytic effect, Cu-BL catalyzes the rapid oxidation of 2,4-DP, presenting a red adduct in the presence of 4-AP and having a strong visible light absorption at 510 nm. Tetracycline drugs can act as inhibitors of this laccase-like activity and reduce the visible light signal at 510 nm. Moreover, due to the internal filtration effect, tetracycline drugs can also quench the blue fluorescence, thereby reducing the fluorescence signal. By combining portable handheld devices and fluorescence capture devices with 365 nm ultraviolet lamps, colorimetric images under visible light related to the concentration of tetracycline drugs can be obtained. These images are processed by smartphone applications, and RGB values are extracted (RGB values are the values of red, green, and blue color channels, which together determine the color information of each pixel in the image), thus providing the analysis results of tetracycline drug concentration. In this paper, a sensitive colorimetric fluorescence dual-mode sensor is designed by using Cu-BL, which has practical significance for the detection of tetracycline drugs.

## 2. Materials and Methods

### 2.1. Synthesis of Cu-NH_2_-BDC and Cu-BL

Cu-NH_2_-BDC was synthesized according to the literature and modified appropriately [32]. An amount of 2 mM of copper nitrate trihydrate (Cu(NO_3_)_2_·3H_2_O) and 1 mM 2-amino-terephthalic acid (NH_2_-BDC) was completely dissolved in 25 mL N, N-dimethylformamide (DMF). The reaction mixture then continued to react at 120 °C for 8 h. After cooling to room temperature, the product was collected, washed, and dried in a vacuum at 60 °C for 24 h. The sample was labeled as Cu-NH_2_-BDC.

To synthesize Cu-BL in the presence of L-lysine, 40 mL of DMF solution was used to dissolve 1 mM of NH_2_-BDC and 2 mM of Cu(NO_3_)_2_·3H_2_O. Then, 80 mL of ethanol (EtOH) and DMF solution were mixed in a 1:1 ratio to dissolve 1 mM of L-lysine. Next, the solution of L-lysine was slowly added dropwise to the previous solution while stirring continuously. Afterward, the mixture was placed in a sealed polytetrafluoroethylene autoclave and heated to 120 °C for eight hours. After cooling to room temperature, it was cleaned and vacuum-dried at 60 °C for 24 h. The sample was labeled as Cu-BL.

### 2.2. Evaluation of Laccase-like Catalytic Activity

The color reaction of phenolic compounds with 4-AP was used to determine the catalytic activities of laccase and the Cu-BL nanoenzyme. The procedure is as follows: MES buffer (30 mM, pH = 7, 700 μL) was mixed with an aqueous solution of 2,4-dichlorophenol (2,4-DP, 1 mg/mL, 100 μL) and an aqueous solution of 4-AP (1 mg/mL, 100 μL). Then, either the laccase solution (1 mg/mL, 100 μL) or the aqueous suspension of Cu-BL was added to the mixture. After one hour of reaction at 60 °C, the reaction mixture was centrifuged at 10,000 rpm for five minutes. Finally, the UV–visible absorbance of the solution was measured at 510 nm. This procedure served as the standard analysis for the duration of the trial unless otherwise noted.

### 2.3. Catalytic Kinetics

To determine the reaction kinetics, 2,4-DP with initial concentrations of 0.05, 0.1, 0.2, 0.4, 0.6, 0.8, 1.0, and 2.0 mM was reacted with 4-AP in the aqueous suspension of Cu-BL in MES buffer. The initial rate of the reaction was calculated by conversion using the Lambert–Beer law (the molar extinction coefficient of the product, 13.6 mM^−1^ cm^−1^) with the Michaelis–Menten equation, shown in Equation (1):

V= V_max_ · [S]/(K_m_ + [S]) (1)

where V is the initial rate, [S] is the substrate concentration, V_max_ is the maximum initial reaction rate, and K_m_ is the Michaelis constant.

### 2.4. Determination of Reactive Oxygen Species (ROS)

To investigate whether reactive oxygen species are involved in the enzymatic catalytic reaction process of Cu-BL, changes in the ultraviolet spectra of the reaction system were observed before and after the addition of reactive oxygen quenchers. Isopropanol was used to quench ·OH, p-benzoquinone was used to quench O^2−^∙, and L-tryptophan was used to quench ^1^O_2_. The experimental conditions for detecting the types of reactive oxygen species in the laccase-like catalytic reaction process of Cu-BL are as follows: different reactive oxygen quenchers (1 mM, 100 μL) were added to the Cu-BL solution. After reacting at 60 °C for 1 h, the absorption spectrum of the reaction system was recorded.

### 2.5. Evaluation of Catalytic Stability

Before evaluating the system’s mimetic enzyme activity, the reaction systems (PBS, MES, and HEPES) were first screened. Based on the above typical experimental process, the effects of pH, temperature, catalyst concentration, phenolic substrates, ionic strength, and organic solvent concentration on the laccase-like activity of Cu-BL and the recyclability of the Cu-BL nanoenzyme were also systematically investigated.

The solutions of 2,4-DP and 4-AP were successively added to different buffers (PBS, MES, HEPES). Subsequently, the aqueous suspension of the Cu-BL nanoenzyme was added to the above solutions. After thorough mixing, the reaction was carried out at room temperature for 30 min, and the color change and UV absorbance value at 510 nm of the solution were recorded.

The catalytic activities of Cu-BL and free laccase were determined within 1 h at 60 °C and pH 3.0–9.0. At the same time, the catalytic reaction was carried out at a temperature of 20 °C to 90 °C and pH = 7.0. In order to study the effect of the Cu-BL nanoenzyme concentration on catalytic activity, different concentrations (0.1–2 mg/mL) of Cu-BL were added. In order to detect the catalytic activity of Cu-BL on different substrates, the aqueous solutions of phenol, p-aminophenol, bisphenol A, 1-naphthol, and p-chlorophenol (1 mg/mL, 100 µL) were determined by the above method.

The catalytic activities of Cu-BL and free laccase were analyzed under the influence of different volumes of ethanol (0, 25%, 50%, 70%, and 100% *v*/*v*) and NaCl solutions with concentrations of 100, 200, 300, 400, and 500 mM. To assess the recyclability of Cu-BL, the aqueous solutions of 2,4-DP, 4-AP, MES buffer, and Cu-BL were allowed to react at room temperature for 30 min. Afterward, they were centrifuged, washed, and then reused for the next reaction cycle.

### 2.6. Colorimetric Detection of TCs

Different concentrations of TCs (0–80 μM) in 100 μL volumes were combined with 100 μL of the 4-AP solution (1 mg/mL) and 700 μL of MES buffer (30 mM, pH = 7). Then, 100 μL of the Cu-BL solution (1 mg/mL) was added to the mixture. After reacting at 60 °C for 1 h, the reaction mixture was centrifuged at 12,000 rpm for 3 min. The absorbance of the supernatant at 510 nm was read and recorded using the photometric measurement mode of the ultraviolet spectrophotometer.

### 2.7. Fluorescence Detection of TCs

Cu-BL has a blue emission peak at 440 nm. The fluorescence intensity of Cu-BL at 440 nm continuously decreases with the addition of different concentrations of TCs. Therefore, a fluorescence sensor based on Cu-BL was constructed. The reaction supernatants of 100 μL of TC solutions with different concentrations after centrifugation were mixed with 2 mL of Cu-BL (1 mg/mL) and static quenching for 1 min. Three parallel experiments were carried out to measure the fluorescence value at 440 nm.

## 3. Results

### 3.1. Catalyst Characterization

**Figure 1:**
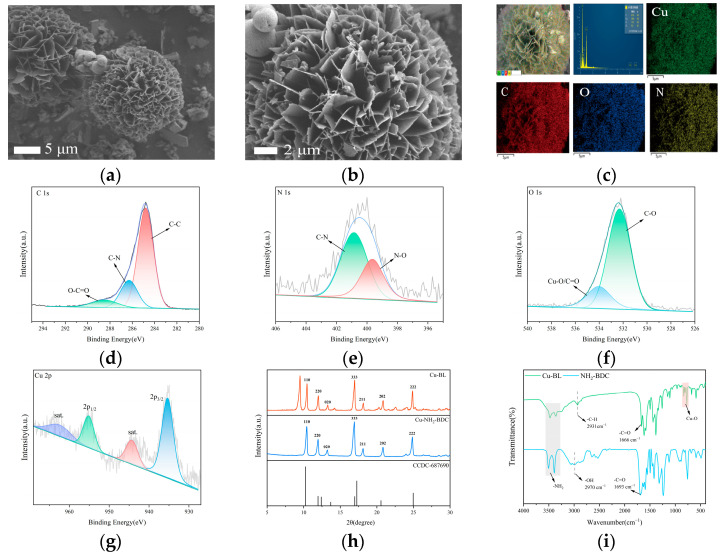
(**a**,**b**) SEM images of Cu-BL at different resolutions; (**c**) EDS elemental mappings of Cu, C, N, and O; (**d**) high-resolution XPS spectra of C 1s, (**e**) N 1s, (**f**) O 1s, and (**g**) Cu 2p; (**h**) XRD patterns; and (**i**) FT-IR spectra.

The morphology of Cu-BL was characterized by SEM. As shown in Figure 1a, Cu-BL as a whole presented a hydrangea-like shape. Observed under higher magnification, its interior was composed of uniform flakes. The results of energy-dispersive spectroscopy (EDS) in Figure 1c showed that elements such as C, N, O, and Cu existed in Cu-BL.

The X-ray photoelectron spectroscopy (XPS) results of Cu-BL are presented as follows: in Figure 1d, the detailed C 1s spectrum is shown, where 288.7 eV corresponds to O-C=O, 286.3 eV to C-N, and 284.8 eV to C-C. Additionally, regarding the N 1s spectrum in Figure 1e, the peak at 399.6 eV is ascribed to N-O, and the peak at 400.9 eV is assigned to N-N. In Figure 1f, for the O 1s spectrum, the two peaks at 532 eV and 533.2 eV correspond to C-O and C=O/Cu-O, respectively. In Figure 1g, the presence of the Cu-O bond in the Cu 2p spectrum suggests the possible coordination of Cu (II) with carboxyl groups. At 935.5 eV and 955.3 eV, they correspond to Cu 2p_3/2_ and Cu 2p_1/2_, respectively. Moreover, the peaks in the ranges of 940–950 eV and 960–969 eV are attributed to the satellite peaks of Cu^2+^, indicating the formation of a single-valence-state framework of Cu^2+^.

The phase purity and crystallinity were analyzed by X-ray diffraction (XRD). As shown in Figure 1h, at 2θ = 10.4°, 12.0°, 13.2°, 16.9°, 18.1°, 20.8°, and 24.8°, they belonged to the (110), (220), (020), (333), (211), (202), and (222) planes (CCDC no. 687690) [33], suggesting that after the loading of amino acid molecules, the parent framework of Cu-NH_2_-BDC was retained and a new crystal phase was formed at 9.5°.

The chemical structure of Cu-BL was characterized by Fourier transform infrared spectroscopy (FTIR). As can be seen from Figure 1i, the absorption peak at 1693 cm^−1^ is the C-O stretching vibration absorption peak of the carboxyl group in Cu-NH_2_-BDC. After coordination with copper, the peak position moved to 1666 cm. The methylene stretching vibration absorption peak of L-lysine in Cu-BL is 2931 cm. In addition, the -OH absorption peak at 2970 cm^−1^ in the ligand disappeared in Cu-BL, which further indicated that Cu(II) and -COOH formed a coordination. The characteristic peaks at 3390 cm^−1^ and 3506 cm^−1^ are related to the symmetric and asymmetric stretching vibration of -NH_2_. In Cu-BL, these peaks shift to lower wave numbers, which are 3361 cm^−1^ and 3479 cm^−1^, respectively, and the absorption peak intensity decreases. The appearance of the Cu-O peak shows that copper ions coordinate with oxygen atoms to form a coordination structure, which echoes the XPS analysis mentioned above.

### 3.2. Evaluation of Laccase-like Catalytic Performance

**Figure 2:**
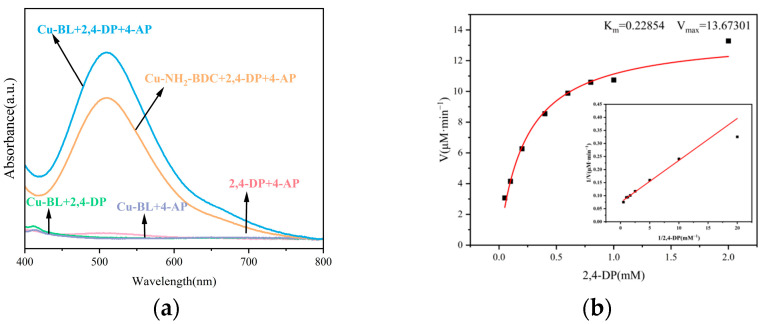

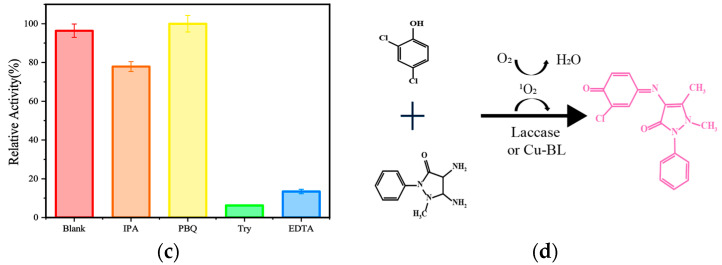
(**a**) UV-vis spectra of different reaction systems. (**b**) Steady-state kinetic analysis of Cu-BL: relationship between the reaction rate and concentration of 2,4-DP catalyzed by Cu-BL. Inset: the corresponding linear Lineweaver–Burk plot of Cu-BL. (**c**) ROS quenching experiment for the oxidation of 2,4-DP by Cu-BL. (**d**) Schematic diagram of the catalytic behavior of Cu-BL.

The laccase-like activity was evaluated by the model color reaction of 2,4-DP and 4-AP. Firstly, a control experiment was conducted to measure the laccase-like activity of Cu-BL. As shown in Figure 2a, 2,4-DP, 4-AP alone, or their mixture had no absorption in the visible area. When either Cu-BL or Cu-NH_2_-BDC was added, a new absorption peak appeared at 510 nm. The results indicate that both Cu-BL and Cu-NH_2_-BDC possess laccase-like catalytic activity, with Cu-BL having relatively higher activity. Batch catalytic reactions were carried out under different conditions to optimize the laccase-like catalytic stability of Cu-BL. Unless otherwise specified, the absorbance of the reaction solution at 510 nm is set as 100%, and the relative activities of other solutions are calculated accordingly.

As shown in Figure 2b, the double-reciprocal (Lineweaver–Burk) curve corresponding to Cu-BL was obtained by fitting the Michaelis–Menten equation, which was 1/V = 0.01607 × 1/[2,4-DP] + 0.0728, with a correlation coefficient R^2^ = 0.985. Based on the fitting results of the Michaelis–Menten equation, the maximum reaction rate Vmax of Cu-BL was 13.67 × 10^3^ mM·min^−1^. Additionally, the Km value of Cu-BL for 2,4-DP was 0.228 M. Compared with other laccase mimetic enzymes (Table 1), Cu-BL generally had good substrate affinity and reaction rate, indicating that the synthesized nanoenzyme had better kinetic performance.

In addition, laccase-like activity may come from the ability of Cu-BL to catalyze the generation of reactive oxygen species (ROS). The ROS method was used to determine the reactive intermediates. Hydroxyl radicals (·OH) could be captured by isopropanol. P-Benzoquinone was used to quench O^2−^·, L-tryptophan was used to quench ^1^O_2_, and EDTA was used as an oxygen vacancy scavenger. Figure 2c shows that there was no significant difference in the absorption of the systems with IPA and PBQ as scavengers compared with that of the group without scavengers, indicating that there were no ·OH and O^2−^·. In the case where L-tryptophan or EDTA was used as a scavenger, the absorption was significantly reduced. Therefore, we determined that ^1^O_2_ and oxygen vacancies were the main reactive intermediates in the laccase-like catalytic reaction. Thus, the laccase-like catalytic behavior of Cu-BL is shown in Figure 2d.

**Figure 3:**
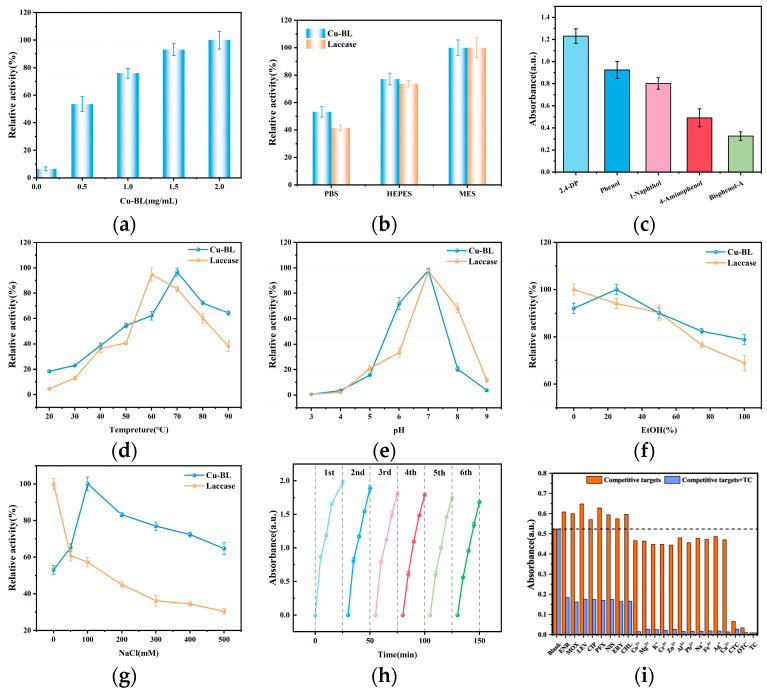
Laccase-like relative activities of Cu-BL and free laccase under different conditions. (**a**) Different concentrations of Cu-BL; (**b**) different buffer solutions; (**c**) for different phenolic substrates, different (**d**) temperatures, and (**e**) pH values. (**f**) In the mixture of ethanol and water; (**g**) different concentrations of NaCl; (**h**) catalytic activities after six cycles; and (**i**) selectivity and anti-interference analysis of Cu-BL detection.

As shown in Figure 3a, when the concentration of Cu-BL is in the range of 0.1–2.0 mg/mL, its catalytic activity increases with the increase in catalyst concentration. However, when considering the amount of catalyst, the activity increases slowly when the concentration is in the range of 1.0–2.0 mg/mL. Therefore, 1.0 mg/mL was selected as the standard dosage. Under the same conditions, we also explored the dependence of Cu-BL on different buffer solutions. As shown in Figure 3b, the catalytic activities of Cu-BL and laccase in the MES buffer solution are higher than those in other solvents. This may be because in MES, 2,4-DP has a relatively high solubility, which helps to improve the effective concentration of reactants and thus accelerate the reaction rate.

Since natural laccase can catalyze various phenolic substrates, the catalytic oxidation ability of the Cu-BL nanoenzyme for phenolic substrates was also tested. The results are shown in Figure 3c. Although the degrees of catalysis differed, the Cu-BL nanozyme could catalyze and oxidize all the aforementioned phenolic substrates, indicating that the Cu-BL nanoenzyme exhibited good substrate universality. Figure 3d shows the catalytic activities in the temperature range of 20–90 °C. For natural laccase, the catalytic activity increased and reached the maximum at 60 °C. For Cu-BL, better catalytic activities could be observed at temperatures ranging from 60 to 80 °C, and the relative activity could remain above 80%. Even when the temperature reached 90 °C, the activity of Cu-BL still remained above 60%. Compared with free laccase, Cu-BL had obvious high-temperature stability at high temperatures. As shown in Figure 3e, as the pH value increased from 3.0 to 7.0, the laccase-like activity of Cu-BL gradually increased, and the optimal activity was achieved at pH = 7.

In view of the common inactivation phenomenon in organic solvents, the laccase-like activity of Cu-BL in ethanol was studied. As shown in Figure 3f, different from natural laccase, when ethanol was in the range of 0–50%, the activity of Cu-BL was higher than that when there was no ethanol in the system. With a further increase in the volume of ethanol, both Cu-BL and natural laccase showed a decline in activity, but the decline rates were different, and the decline of Cu-BL was relatively mild.

The catalytic activity in a certain concentration of NaCl solution is an important parameter for the practical application of the nanoenzyme. As shown in Figure 3g, with the increase in sodium chloride concentration, the catalytic activity of natural laccase decreased obviously, which may be due to the inactivation of natural laccase by chloride ions. In contrast, Cu-BL exhibited stronger catalytic activity with the increase in ionic strength. This might be because the presence of salt increased the ionic strength of the solution and generated a salting-out effect [35], thus promoting the catalytic performance of Cu-BL.

Furthermore, as shown in Figure 3h, in the repeated recycling experiment, the Cu-BL nanozyme was recovered by centrifugal separation and still maintained a relative activity of approximately 80% after six cycles of repeated catalysis, while the free natural laccase could not be recycled.

The above control experiments consistently demonstrated that compared with natural laccase, Cu-BL had good catalytic stability in the presence of high-temperature organic solvents and sodium chloride.

In the detection of actual samples, high selectivity is the key factor to measure the feasibility of the analysis and detection platform. Under the same conditions, metal ions and common antibiotics (ENR, MOX, LEV, CIP, PEX, NIS, ERV, CHL) were used to evaluate the selectivity and anti-interference ability of the TCs/Cu-BL nanoenzyme sensing system. As shown in Figure 3i, due to the similar structures of TC, CTC, and OTC, the absorption value of the TCs/Cu-BL nanoenzyme sensing system is similar at 510 nm. However, they are rarely added to the same drug at the same time, so they do not interfere with each other when tested separately. In addition, the change in absorbance in the presence of other competitive antibiotics is much lower than that in the sensing system containing TCs. In the TCs/Cu-BL nanoenzyme sensing system, after adding other interfering substances additionally, it was found that there was no significant change in absorbance in the system with added metal ions, while the absorbance increased in the system with antibiotic interference but there was still a significant gap compared with the Cu-BL nanoenzyme sensing system. Therefore, it indicated that the proposed system could detect the three TCs with high specificity and had good anti-interference ability.

### 3.3. Colorimetric Detection of TCs Performance of TCs Detection with Colorimetry

**Figure 4:**
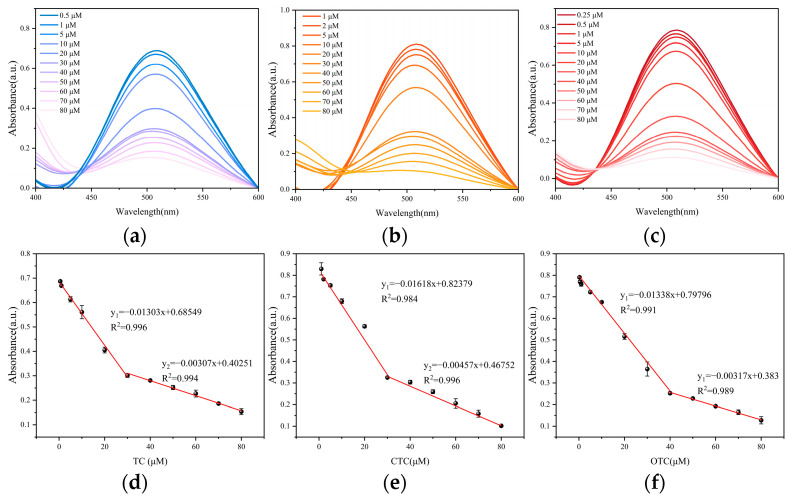
UV–visible absorption spectra of the Cu-BL nanoenzyme sensing system under the action of different concentrations of (**a**) TC, (**b**) CTC, and (**c**) OTC. (**d**–**f**) Calibration curves of the change in absorbance versus the amount of TCs added.

In order to evaluate the ultraviolet detection performance of TCs, the ultraviolet–visible absorbance of different concentrations of TCs at 510 nm was measured. As shown in Figure 4a–c, because of their similar chemical structures, their responses to these three TCs are similar. With the increase in the concentration of TCs, the ultraviolet absorbance at 510 nm gradually decreased. As shown in Figure 4d–f, there was a linear relationship between the absorbance of the Cu-BL detection system at 510 nm and the concentration of TCs. As the dose of TC increased, the color change could also be seen with the naked eye, gradually changing from pink to colorless. The linear ranges of the ultraviolet absorbance measured for TC, CTC, and OTC were 0.5–30–80 µM, 1–30–80 µM, and 0.25–40–80 µM, respectively, and all exhibited a two-segment linear relationship. It is speculated that in the first low-concentration range, the active sites of the Cu-BL nanoenzyme were relatively sufficient, and the binding probability of each TC molecule to the active sites of the nanoenzyme was relatively high. After TCs reached the second high-concentration range, the TC molecules bound to the nanoenzyme were close to saturation, and the competitive inhibition effect on the substrate gradually weakened, resulting in a slower decrease in absorbance. At the same time, based on the three-sigma rule (S/N = 3), the limits of detection (LOD) for TC, CTC, and OTC were 0.27 µM, 0.22 µM, and 0.26 µM, respectively. These results strongly indicate that the Cu-BL detection system has a satisfactory and relatively wide linear range under low-concentration conditions, thereby showing potential for practical application. When compared with other detection methods, the Cu-BL detection system performs similarly or better. Although some reported methods exhibit higher sensitivity or a wider linear range, our method features a simpler analysis process, lower cost, and reliable analysis results.

### 3.4. Fluorescence Detection of TCs Performance of TCs Detection with Fluorometry

**Figure 5:**
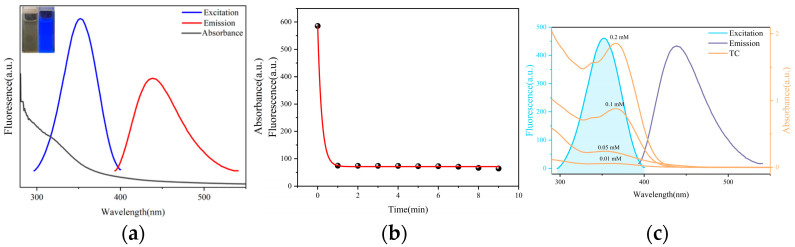

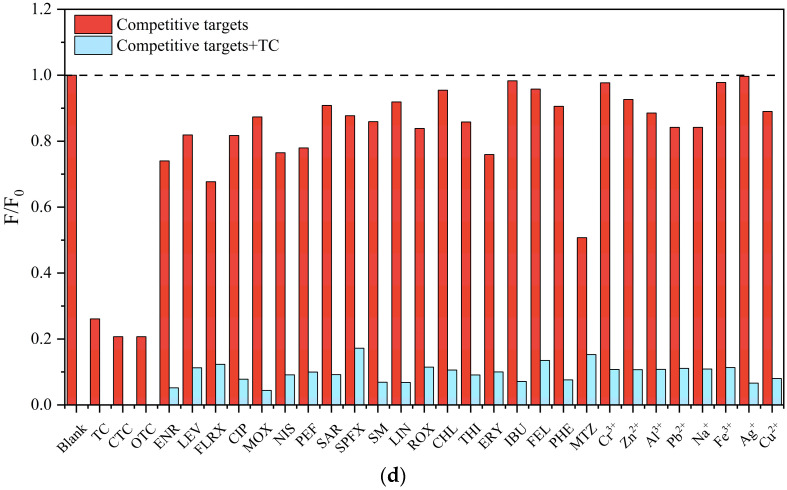
(**a**) Fluorescence excitation spectrum, emission spectrum, and ultraviolet absorption spectrum of Cu-BL. The inset shows the fluorescence image under a 365 nm ultraviolet lamp. (**b**) Change in the fluorescence intensity of Cu-BL within 10 min after adding TC. (**c**) Fluorescence excitation spectrum, emission spectrum, and ultraviolet absorption spectra of different concentrations of TC for Cu-BL. (**d**) Investigation of the selectivity and anti-interference ability of the fluorescence detection of Cu-BL.

Previous studies [36] showed that NH_2_-BDC, a photoluminescent organic molecule, endows the obtained MOFs UIO-66-NH_2_ MOF with fluorescence properties. We hypothesized that the prepared Cu-BL with NH_2_-BDC as the ligand might have fluorescence properties. As expected, as shown in Figure 5a, the obtained Cu-BL exhibited fluorescence emission at 440 nm, like the pure ligand NH_2_-BDC. The inset shows that under the irradiation of a 365 nm ultraviolet lamp, there was bright blue fluorescence in the Cu-BL aqueous solution, clearly confirming its photoluminescence.

In this experiment, the photostability of the Cu-BL water suspension was examined. As can be seen from Figure S1, the Cu-BL water suspension maintained excellent stability when continuously irradiated for 60 min. Meanwhile, this experiment also investigated the impact of Cu-BL water suspensions with different concentrations on fluorescence intensity. The measurement results, as shown in Figure S2, indicate that changes in concentration did not significantly affect the fluorescence intensity. Furthermore, to explore the stability of Cu-BL during the detection of tetracycline (TC), we studied the fluorescence changes in the Cu-BL + TC system within 10 min. As can be observed from Figure 5b, the fluorescence value dropped to the lowest point within 1 min after adding TC and remained stable for the subsequent 10 min. This result also demonstrates that this method exhibits the advantages of rapid response and stable reliability when used for the fluorescence detection of TC.

Fluorescence quenching mainly includes static quenching [37], dynamic quenching [38], fluorescence resonance energy transfer (FRET) [39], photo-induced electron transfer (PET) [40], and the inner filter effect (IFE) [41]. To check the fluorescence quenching mechanism, the ultraviolet absorption spectra of TC solutions with different concentrations were recorded and compared with the fluorescence spectrum of Cu-BL. As shown in Figure 5c, the absorbance of TC at 350 nm had a good overlap with the fluorescence spectrum of Cu-BL. As the TC concentration increased, the overlapping proportion expanded, indicating that the quenching effect might be due to the inner filter effect (IFE) [42].

Before systematically detecting TCs, metal ions such as Cr^3+^, Zn^2+^, Al^3+^, Pb^2+^, Na^+^, Fe^3+^, Ag^+^, and Cu^2+^, as well as Enrofloxacin (ENR), Levofloxacin (LEV), Fleroxacin (FLRX), Ciprofloxacin (CIP), Moxifloxacin (MOX), Norfloxacin (NOR), Pefloxacin (PEF), Sarafloxacin (SAR), Sparfloxacin (SPFX), Roxithromycin (ROX), Erythromycin (ERY), Chloramphenicol (CHL), Thiamphenicol (THI), Streptomycin (STR), Lincomycin (LIN), Ibuprofen (IBU), Felodipine (FEL), Phenacetin (PHE), and Metronidazole (MTZ), were, respectively, used to verify the selectivity and anti-interference ability of the TCs/Cu-BL nanozyme fluorescence sensing system. As shown in Figure 5d, only the fluorescence intensity of the target TCs changed significantly, while the changes in all the detected interfering substances were negligible. These results indicate that the prepared fluorescence sensor has high selectivity and anti-interference ability for TCs and shows great potential for practical applications.

**Figure 6:**
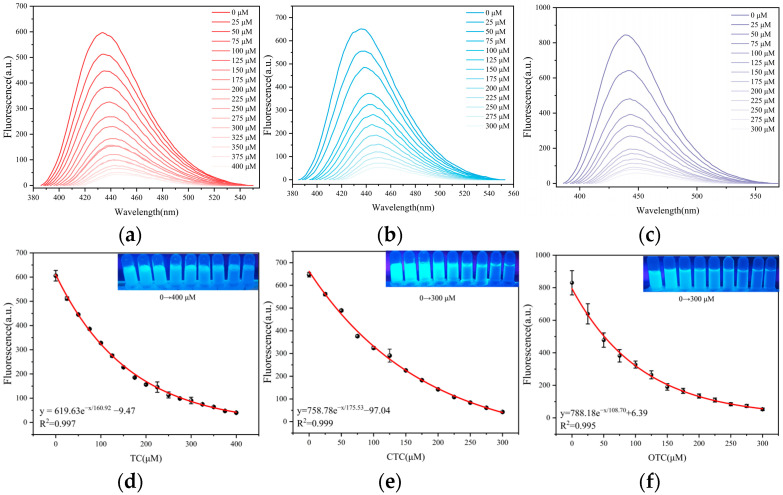
(**a**–**c**) F440 nm fluorescence emission spectra of the Cu-BL nanoenzyme sensing system under the action of different concentrations of (**a**) TC, (**b**) CTC, and (**c**) OTC. (**d**–**f**) Calibration curves of the change in fluorescence intensity versus the amount of TCs added. The inset shows the fluorescence images of different concentrations of TCs under a 365 nm ultraviolet lamp.

As shown in Figure 6a–c, in the mixed solution of Cu-BL and TCs, the fluorescence intensity at 440 nm (F440 nm) decreased significantly with the increase in TC concentration. It could be observed with the naked eye that as the concentration of TCs increased, the obvious fluorescence gradually weakened until there was almost no fluorescence. The corresponding calibration curves are shown in Figure 6d–f. The concentrations of TCs and the fluorescence absorption intensities all conformed to the ExpDec1 model (y = A× exp(−x/t) + y_0_). Among them, the linear range of TC was 0–400 µM with a correlation coefficient of 0.997; the linear range of CTC was 0–300 µM with a correlation coefficient of 0.999; and the linear range of OTC was 0–300 µM with a correlation coefficient of 0.995.

**Figure 7:**
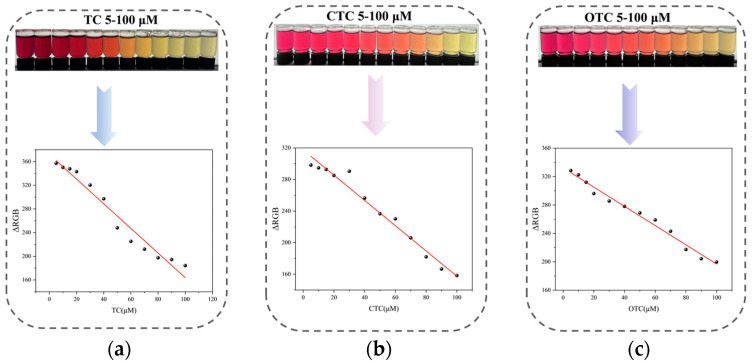
Smartphone visual analysis of (**a**) TC, (**b**) CTC, and (**c**) OTC based on Cu-BL.

The rapid analysis and visualization of TCs are crucial for environmental monitoring and food safety control. Therefore, a more portable visual analysis method based on smartphones, integrating colorimetric and fluorescence modes, was developed. The smartphone’s camera captured the color changes in the Cu-BL detection system with different concentrations of TCs added (Figure 7). The Colorimetric Titration software extracted the corresponding RGB values from the images. In the range of 5–100 μM, the ΔRGB values were linearly related to the concentrations of TCs, and the regression equations were y_1_ = −2.08 C_TC_ + 372.14 (R^2^ = 0.9607), y_2_ = −1.60 C_CTC_ + 317.52 (R^2^ = 0.9739), and y_3_ = −1.60 C_OTC_ + 332.31 (R^2^ = 0.9842).

## 4. Conclusions

In summary, we successfully synthesized a copper-based bionic laccase with NH_2_-BDC and L-lysine as dual ligands, and this enzyme exhibits fluorescence properties. The designed Cu-BL showed a smaller Km value (0.228 mM) and higher Vmax (13.67 × 10^−3^ mM min^−1^) than early reported results. Subsequently, based on the inhibitory effect of TCs on the reaction between 2,4-DP and 4-AP, colorimetric methods were established with linear ranges for TC, CTC, and OTC of 0.5–30–80 µM, 1–30–80 µM, and 0.25–40–80 µM, respectively, and limits of detection (LODs) of 0.27 µM, 0.22 µM, and 0.26 µM. Furthermore, since tetracycline drugs (TCs) can quench the fluorescence of the Cu-BL nanoenzyme through the internal filtration effect (IFE), fluorescence methods were also developed with linear ranges for TC, CTC, and OTC of 0–400 µM, 0–300 μM, and 0–300 μM, respectively. The platform proposed for TC sensing features a “switch-off” signal response and high sensitivity. Overall, the colorimetric/fluorescent dual-mode sensing platform we proposed can be used for the simultaneous detection of various tetracycline antibiotics. This strategy is simple to operate, enables rapid detection, provides reliable results, and is cost-effective.

## Figures and Tables

**Table 1:** A comparison of laccase-like catalytic kinetic parameters of Cu-BL with other free laccase and laccase simulators reported before.

Catalyst	K_m_ (mM)	V_max_ (mM min^−1^)	Reference
Cu-BL	0.228	13.67 × 10^−3^	This work
Laccase	0.5388	2.4159 × 10^−4^	[23]
Cu-NH_2_-BDC-Mel	0.1877	1.79 × 10^−3^	[23]
CH-Cu	0.42	7.32 × 10^−3^	[14]
CN-MOF-818	4.481	3.7650 × 10^−3^	[34]

## Data Availability

Data are contained within the article and Supplementary Materials.

## Supplementary Materials

The following supporting information can be downloaded at: https://www.mdpi.com/article/10.3390/nano15030162/s1, Figure S1. Photostability of Cu-BL aqueous suspension under continuous illumination for 60min. Figure S2. Effect of different concentrations of Cu-BL aqueous suspension on fluorescence intensity.
