# Supplementary material for: A Colorimetric and Fluorescent Dual-Mode Sensor Based on a Smartphone-Assisted Laccase-like Nanoenzyme for the Detection of Tetracycline Antibiotics
Source: Nanomaterials (Basel). 2025 Jan 22;15(3):162. doi: 10.3390/nano15030162 (PMC11820295; doi:10.3390/nano15030162)
Supplement: Supplementary file 1 [file nanomaterials-15-00162-s001.zip › nanomaterials-3425067-supplementary.pdf]

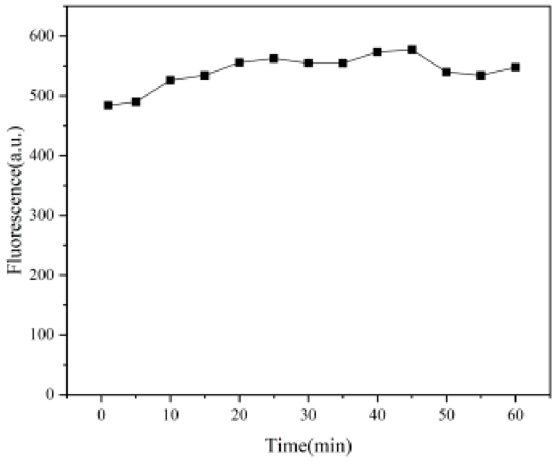

S1.Photostability of Cu-BL aqueous suspension under continuous illumination for 60min

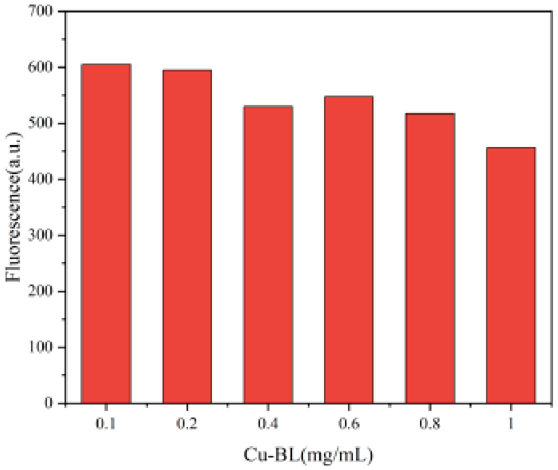

S2.Effect of different concentrations of Cu-BL aqueous suspension on fluorescence intensity
